# Ninth Pharmacologic-Historical Forum, 2024, Munich, Germany: the development of experimental pharmacology in Munich at the Walther Straub Institute

**DOI:** 10.1007/s00210-024-03338-7

**Published:** 2024-08-03

**Authors:** Peter Eyer

**Affiliations:** Walther Straub Institute of Pharmacology and Toxicology, Ludwig Maximilian University, Goethestraße 33, 80336 Munich, Germany

**Keywords:** Peter Eyer, August Wilhelm Forst, Wolfgang Forth, Thomas Gudermann, Manfred Kiese, Max Pettenkofer, Walther Straub, Hermann Tappeiner, Carl Voit

## Abstract

In 1887, Hermann Tappeiner (1847–1927) was appointed as professor for medicinal chemistry and pharmacology. He studied the role of intestinal bacteria and contributed to better understanding of digestion. In 1923, Walther Straub (1874–1944) succeeded. He was at the zenith of his scientific career, gained habilitation in Leipzig already in 1900, accepted the direction of the Institute of Pharmacology at Marburg in 1905, of Würzburg in 1906, before he moved to Freiburg in 1907. Straub preferred quantitative studies with various alkaloids, cardiac glycosides, and Senna glycosides on isolated organs. One important legacy is his contribution “Die Digitalisgruppe” in Hefters Handb. Exp. Pharmakol. 1924. Walther Straub was editor of Naunyn–Schmiedeberg’s Archives of Pharmacology and founded the Deutsche Pharmakologische Gesellschaft in 1920. In 1944 when most of the institute was destroyed by air raids, Walther Straub retired and succumbed in Bad Tölz. In 1946, August Wilhelm Forst (1890–1981), a pupil of Straub, was appointed to head the institute ruins. We owe to him the provisional reconstruction of the old building, institution of an Insulin Control Laboratory, and the development of a vibratory cage that allowed the registration of psychomotor activity in rodents. Forst published the first comprehensive review on “Detoxication.” In 1961, Manfred Kiese (1910–1983), a pupil of W. Heubner, came from Tübingen and accompanied the erection of a new building. Kiese made important contributions to the understanding of the biotransformation of foreign compounds and was the first to describe the biological N-oxygenation. His studies on ferrihemoglobin formation resulted in the development of an effective cyanide antidote, 4-dimethylaminophenol. “Methemoglobinemia, a Comprehensive Treatise” is part of his scientific legacy. In 1980, Wolfgang Forth (1932–2009) from Bochum headed the institute and convinced the medical faculty of LMU to rename the building into Walther Straub Institute. His scientific interests were centered on interactions between essential and toxic metals during intestinal absorption. He was co-editor of the German Textbook on Pharmacology and Toxicology founded in 1975, which is presently in its 13th edition. In 2000, Peter Eyer (1942) was commissioned to lead the institute until Thomas Gudermann (1960) was appointed to direct the chair in 2008.

## Introduction

This overview presents the written version of a lecture given at the Ninth Pharmacologic-Historical Forum on the occasion of the 9^th^ German Pharm-Tox Summit held on 13–15 March 2024 in Munich. The evolution of experimental pharmacology and toxicology in Munich has a history of some 140 years when the first chair was established at Ludwig Maximilian University in 1883. Meanwhile, the metropolitan area of Munich counts at least 10 separate institutions dealing with pharmacology and toxicology. The present overview, however, restricts to the history of the Walther Straub Institute where the seeds of experimental pharmacology had begun to sprout. (For more details, particularly regarding appointed lecturers, their fate, and major focus of scientific work, the reader may be referred to a former article (Eyer 2004).)

## Initial reluctance of most European universities to establish chairs of pharmacology

The pioneering work of François Magendie (1783–1855) in Paris and Rudolph Buchheim (1820–1879) in Dorpat, who are generally regarded as the founders of experimental pharmacology, did not encourage other medical faculties in Europe to create separate chairs of pharmacology in the first half of the nineteenth century (Muscholl 1995). In contrast, the discipline of pharmacology was held superfluous for medical students as the famous surgeon in Vienna, Theodor Billroth, denounced in 1876: “The formerly given lecture on Materia Medica is nowadays disassembled into pharmacology, pharmacognosy, medication prescription, and toxicology. It is of course difficult to employ a full professor sufficiently, if he teaches only this single discipline, since the content of such a lecture has considerably shrunk and the remainder could be easily halved. The lecture in pharmacology should restrict to a brief overview of the most important groups of drugs and demonstrate the action of the most toxic substances. This can be accomplished in 3–4 semester hours. Students should not be burdened with surplus lectures on this topic.” (Billroth 1876).

**Fig. 1:**
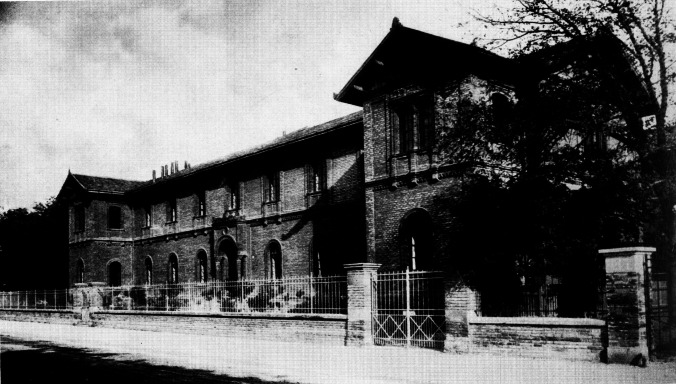
Institute of Physiology at Findlingstraße 12, built between 1855 and 1857 (Archive of Walther Straub Institute)

When the Ludwig Maximilian University moved from Landshut to Munich in 1826, Materia Medica was taught by an anatomist or physiologist, depending on personal preference and expertise, and it took another 40 years until the physiologist Carl Voit and the Hygienist Max Pettenkofer prepared the fertile soil for an experimentally based pharmacology (Goerke 1972). In fact, it was the hospitality of Carl Voit who offered 4 rooms to Hermann Tappeiner for pharmacological experiments in the new Institute of Physiology at the then Findlingstraße 12, nowadays Pettenkoferstraße. The building, outside the city boundaries with an unspoilt view of the Alpes, was erected in the style of mountain farmhouses with vastly cantilevered roofs (Fig. 1) (Wildi-Torster 1997). Here, Hermann Tappeiner found shelter when he was appointed adjunct professor (*Extraordinarius*) for Medicinal Chemistry and Pharmacology at the Ludwig Maximilian University of Munich in 1883, but without endowment of personal laboratories. Hence, we consider Hermann Tappeiner as the founder of experimental pharmacology in Munich.

Notwithstanding, there had been some germinal ancestors in Munich, who slightly touched pharmacological topics: e.g., the pharmacist Johann Andreas Buchner, who isolated salicine, the glucoside of saligenin, from the bark of willow in 1828, while his son Ludwig Andreas Buchner succeeded in getting the chair of pharmacy and toxicology in 1852. Both were occasionally depicted as pharmacologists although not adhering to our modern understanding of pharmacology, in that an experimental analysis of the drug effects proper was still lacking. The same holds true for a medicinal predecessor: the physician Hermann von Boeck who was appointed lecturer in pharmacology in 1871 with his thesis “On the degradation of proteins in the animal body by morphine, quinine and arsenous acid.” In 1876, he obtained an extraordinary professorship and published a 250-page paper on “Intoxications with poisonous plants” in the Handbook of Special Pathology and Therapeutics edited by Hugo von Ziemssen. However, he received no position at the university and had to earn his livelihood as a practitioner. A chronic liver disease finished his life early. Hence, it is Hermann Tappeiner who should be considered as the first pharmacologist in Munich (Goerke 1972).

## Hermann Tappeiner

**Fig. 2:**
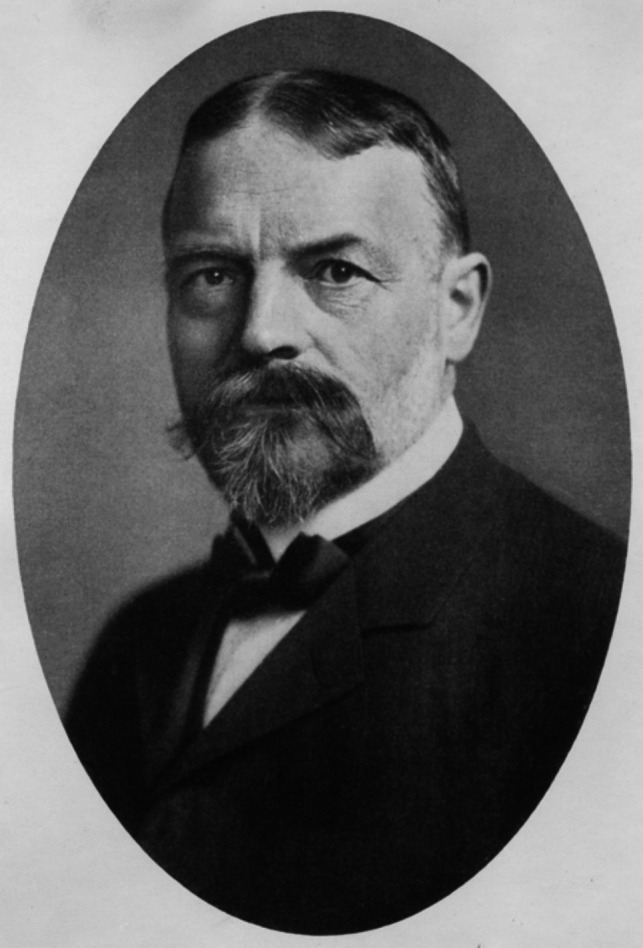
Hermann Tappeiner (about 1900) (Archive of Walther Straub Institute)

Hermann Tappeiner (Fig. 2) was born in Meran on November 18, 1847, as the son of the famous anthropologist and expert in tuberculosis Franz Tappeiner who encouraged the construction of the well-known Tappeiner promenade. After medical studies in Innsbruck, Göttingen, Leipzig, Heidelberg, and Tübingen, Hermann Tappeiner received his doctorate in Leipzig with an experimental work on “Consequences for the blood stream after ligation of the portal vein.” In Munich, he was appointed lecturer for Medicinal Chemistry at the physiological institute of Carl Voigt in 1877 with the title: “Oxidation of cholic acid by acidic potassium chromate in sulphuric acid.”

**Fig. 3:**
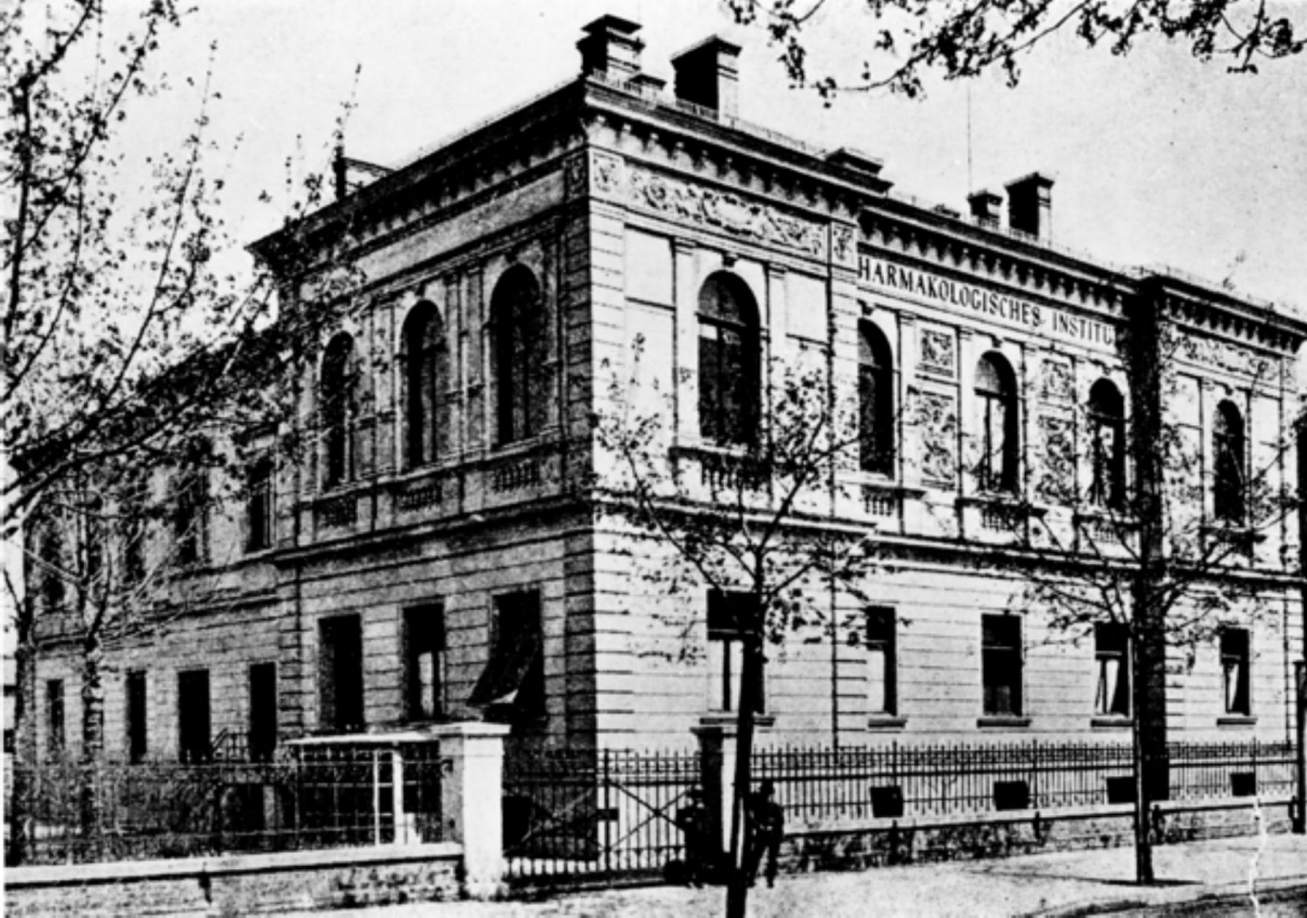
The first Institute of Pharmacology at Nussbaumstraße 28, Munich (1893) (Archive of Walther Straub Institute)

In 1879, he accepted the chair as professor of physiology and dietetics at the Royal Veterinary School in Munich that did not yet belong to the university. Since the experimental facilities had been rather poor there, he gladly received his appointment as an extraordinary professor of Medicinal Chemistry and Pharmacology at the medical faculty of the Ludwig Maximilian University. Finally, in 1893, he was appointed full professor and received a new building at Nussbaumstraße 28 in Munich (Fig. 3).

Upon opening ceremony, Tappeiner sketched out the tasks of pharmacology: “In the last decades the Materia Medica was swollen to a mess of natural and synthetic products. Only a small part of it had been sufficiently approved bedside. Most of it had been uncritically used upon mystic and natural philosophical speculations or superficial experience and blind belief in authority”… “In contrast, modern pharmacology explores the mode of actions elicited by chemical substances in the organism and contributes to the advances of physiology. Moreover, chemical changes that drugs and foreign compounds undergo in the body have widened our view on the chemical processes of life.” He proceeded: “Admittedly, even today pharmacology has detected only macroscopically the targets of drug actions and is able to present a kind of topology of drug effects. The molecular events, however, are still obscure and await future elucidation” (Tappeiner 1893). No doubt, this kind of view characterizes Hermann Tappeiner as a prototype of modern pharmacology.

During his 36-year direction, Tappeiner studied the role of intestinal bacteria, particularly in ruminants, and contributed to a better understanding of digestion and enteral absorption. Together with his pupil Oscar Raab, he was interested in mechanisms of photodynamic and phototoxic reactions. In addition, Tappeiner became known through his instructions of chemically based bedside diagnostics and his textbook of Pharmacology and of Medication Prescription, which appeared in 1885 and 1890, respectively. Tappeiner passed away on January 12, 1927, in Munich. He was married to Elisabeth von Ziemssen since 1882; the couple had 4 children (Forth and Klimmek 1994).

## Walther Straub

**Fig. 4:**
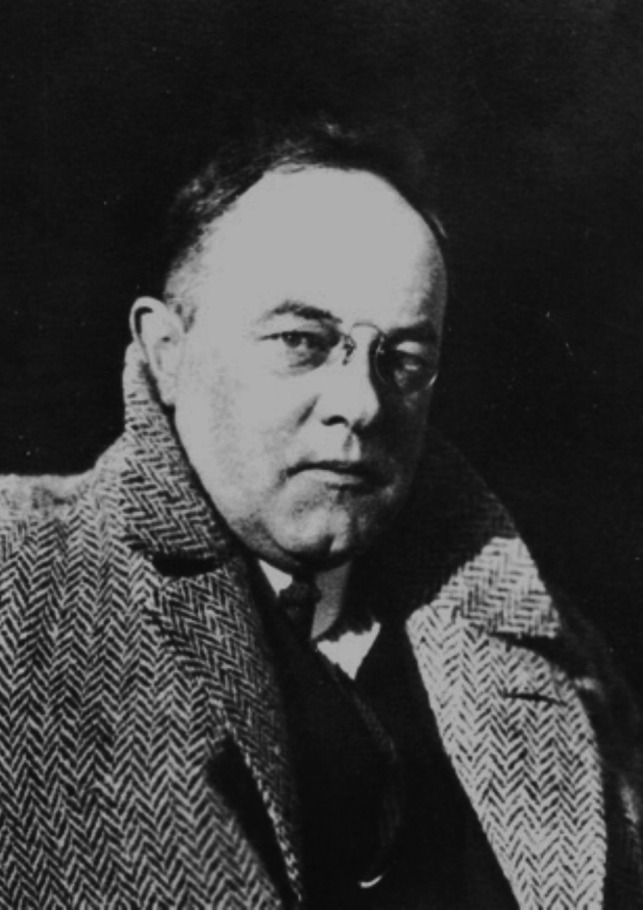
Walther Straub 1924 (Archive of Walther Straub Institute)

**Fig. 5:**
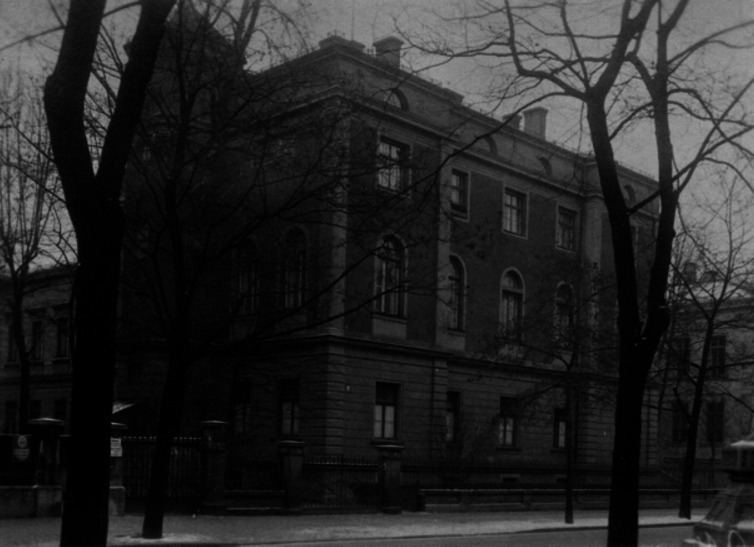
The enlarged Institute of Pharmacology at Nussbaumstraße 28, Munich (1932) (Archive of Walther Straub Institute)

**Fig. 6:**
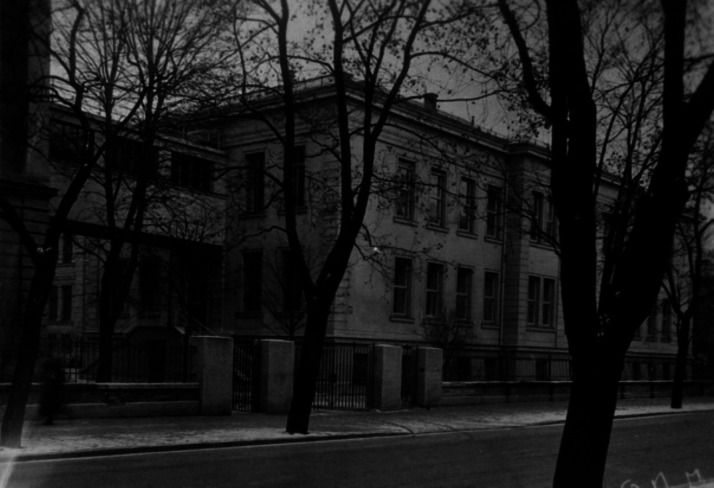
The former Institute of Pathology at Nussbaumstraße 26 connected to the Institute of Pharmacology via a 13-m-long bridge, called “*Straub’sche Brücke* “ (Archive of Walther Straub Institute)

In 1919, an appointment committee was headed by the then dean of the Medical Faculty, Ferdinand Sauerbruch to search for a successor for the 72-year-old Tappeiner. The faculty decided to appoint *unico loco* Walther Straub from Freiburg where he had held the chair of pharmacology for over 16 years (Fig. 4). After extensive negotiations and abundant correspondence with Sauerbruch and later Otto Frank, Straub accepted. He was promised the rebuilding of the Institute of Pharmacology in which a flat for Walther Straub and his family was erected on the uppermost floor (Fig. 5). Moreover, the neighboring building of the Institute of Pathology at Nussbaumstraße 26 was assigned to the Institute of Pharmacology. Straub arrived in Munich on April 1, 1923. It took until 1932 when the rebuilding of both institutes was completed. The old Institute of Pharmacology was enlarged and combined with the former Institute of Pathology via a 13-m-long bridge made of ferroconcrete and called *Straub’sche Brücke* (Goerke 1972) (Fig. 6).

When Straub came to Munich, he was at the zenith of his scientific career and was acknowledged as one of the most important pharmacologists of the first decades of the twentieth century in Germany and even in Europe. In 1920, he provided the impetus for founding the German Pharmacological Society (*Deutsche Pharmakologische Gesellschaft*) and became the editor of the oldest journal of pharmacology: the *Naunyn-Schmiedebergs Archiv für experimentelle Pathologie und Pharmakologie*. In 1925, the conference of the German Pharmacological Society was held in Rostock under the heading “Straubismus convergens”—an ironic allusion to the term “*strabismus convergens*” as pointed out by Walther Siegfried Loewe, Dorpat (Forst 1974).

Born in Augsburg on May 8, 1874, Straub studied medicine in Munich, Tübingen, and Straßburg where he as a medical student started some experimental work under Oswald Schmiedeberg on glycosuria following carbon monoxide poisoning, which was published in 1897 (Straub 1897). After completion of his medical study in Munich, he worked on his thesis under Carl Voit in the Department of Physiology and received his degree as *Dr.med.* on July 18, 1897, which was published later under the title: “The influence of sodium chloride on the decomposition of proteins” (Straub (1899). When he became an assistant of Carl Voit, he made the acquaintance with his later friend Otto Frank before he left Munich to enter the laboratories of pharmacology at Leipzig University headed by Rudolf Boehm, the second famous foster father in pharmacology. Within 2 years, he qualified on July 23, 1900, as a Lecturer in Pharmacology with a study on the action of Antiarin on the isolated suspended frog heart. (Antiarin was used as arrow poison due to its digitalis-like properties (Straub 1900).) Immediately afterwards, he visited the Zoological Station at Neaples, where he had the opportunity to use marine animals, particularly *Aplysia limacina*, a sea snail with a heart devoid of regulatory innervation that was considered to allow physiological studies on the smooth musculature proper. (For details, the reader is referred to the comprehensive review of Otto Krayer in the edition of Melchior Reiter (1998).)

Not yet a full professor, Straub in 1905 was appointed to the chair of pharmacology in Marburg, 1 year later he followed a call to Würzburg and relinquished this position in 1907 when he was given the opportunity to become the first full professor of pharmacology at the University of Freiburg. Between 1913 and 1917, a new institute was built, and a very fruitful period started when Straub gathered some very talented coworkers who later obtained chairs at several German universities. (For details, see Starke 2007.)

**Fig. 7:**
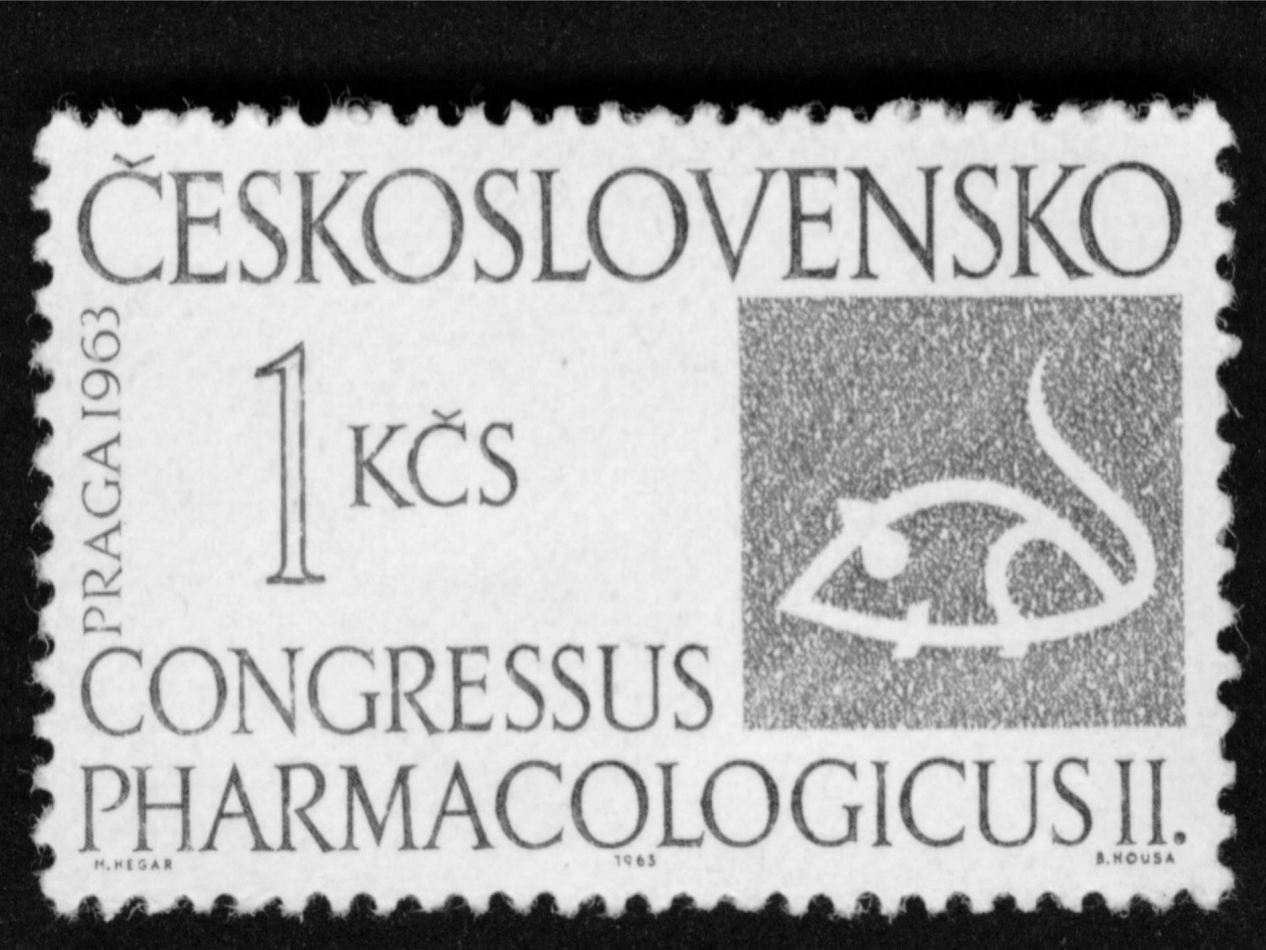
Straub’s Mouse-Tail-Phenomenon depicted on a Czech stamp that was delivered on occasion of the Second International Congress of Pharmacology held in Prague 1963

Much has been written on the scientific oeuvre of Walther Straub (for details, see Krayer and Reiter 1998; Prüll et al. 2009). His name relates to an experimental design using the isolated frog heart (“Straub’s Frog Heart”) and to “Straub’s Mouse-Tail-Phenomenon” a bioindicator for morphine-like substances. This phenomenon has been described as catatonic rigidity of the tail that is maximally deflected parallel to the backbone (Straub 1911). By chance, this phenomenon was observed when the newly synthesized pethidine, designed as a possible anticholinergic candidate, was tested in mice for toxicity. From then, pethidine was classed as a morphine-like drug (Schauman 1940). On the occasion of the Second International Congress of Pharmacology held in Prague 1963, a stamp was disseminated showing Straub’s Mouse-Tail-Phenomenon (Fig. 7).

Most attention Straub devoted to the mode of action of digitalis glycosides, which were studied in a large kind of differently developed animals. He summarized the pertinent knowledge in his ample legacy (Straub 1924). The particular merits of his experimental approach center on the quantitative relationship between drug effect and drug concentration. He developed many bioassays to quantify the amount of drug that had been absorbed by a selected tissue. In doing so, he had his own views on the mode of drug action that contrasted with the receptor concept as advanced by Ehrlich and Langley (for details, see Prüll et al. 2009).

During his decades in Munich, Straub extended his investigations to the smooth muscles of the intestine and to questions of intestinal transport, and he characterized the mode of action of Senna glycosides.

An appreciation of Walther Straub would be incomplete if his many statements about contemporary issues would be ignored. Already before WW I, Straub was eager to immunize practitioners against intolerable advertising promises of pharmaceutical manufacturers. Thus in 1908, he denounced the intense advertising of a new antipyretic/analgesic drug “Pyrenol” that consisted of sodium benzoate, thymol, and salicylic acid “in a particular physico-chemical bonding relationship.” Straub pulled the blade: “The manufacturer shows a constitution formula of his product with a fairytale-like connecting hyphen that has only literary meaning. Chemically, this tied construct does not really exist, but a chemical formula is always impressive, and the addressee is clueless and stupid. You could even claim this was the formula of uric acidic glucose”… “It’s a shame that the many addressees do not realize the fakes and have no longer any knowledge of pharmacology. This shows us that something went wrong and where an improvement is indispensable. The defects are due to our medical teaching and exams, where theoretical aspects are missing out. The major educational goal in pharmacology appears to be the correct medical prescription of drugs” (Straub 1908).

On other occasions, Straub was a brilliant writer, too, who could hide a lot between the lines. In 1943, Straub, a passionate coffee drinker, submitted an article, wisely in Swiss Medical Weekly (*Schweizer Medizinische Wochenschrift*), on “Coffee needs then and now” (*Kaffeenöte einst und jetzt*). Herein, he was sarcastic about the coffee substitutes, which during the war were advertised to the Germans: “These substitutes differ from coffee proper in that they introduce some nutritional compounds, but do not elicit enjoyment at all. From a standpoint of kitchen technology, you should call it flower soup, but not delicacy” (Straub 1943).

The oppressive time of National Socialism was not without impact on the Pharmacological Institute of the Munich University. Straub had trouble with the Nazi authorities already in 1933 when he supported his first assistant August Forst who refused to raise the flag with swastika on the roof of the institute when Straub was in the USA. Subsequently, Straub had to struggle for the employment of Forst who was steadfast to remain with his half-Jewish wife, which resulted in the interdiction of exams and refusal of the promotion to an extraordinary professor. In 1935, Straub wrote to the Directorate of the University: “The Institute of Pharmacology urgently needs a third assistant, because the lectures of pharmacology have to be enlarged to also teach pharmacists due to the new curriculum. Since the Private Docent Forst is not allowed to hold exams, a substitute is required who can only be an assistant of the institute.”

When Straub publicly criticized amateurish concepts of the responsible Police Inspection how to treat phosphorus burns, he hardly escaped an impeachment during WW II. Straub had detected already in 1903 that CuSO_4_ was able to inactivate yellow or white phosphorus thus inhibiting self-ignition. His recipe of 1% CuSO_4_ in a paste of bolus alba was well known to lay press as “*Straubsche Paste*” and helped to refrain the ill-reputed phosphorus burns from creepiness (“*und half die so übel beleumdeten Phosphorbrandwunden zu entgruseln*”; Straub 1944).

The irony of fate would have it that by the air raids in July 1944 three “cans,” i.e., phosphorus-kindled incendiary bombs ignited the roof-truss of the old building of the former Institute of Pathology, before high-explosive bombs in a later air-raid did the rest. Contemporaries reported that Walter Straub, sitting across the street under a tree, had been watching how the building was burning down. Shortly thereafter, Straub succumbed in Bad Tölz to a fatal stroke on October 22, 1944 (Forth 1994).

Straub had become a member of the *Deutsche Akademie der Naturforscher Leopoldina* in 1925, an honorary member of the American Society for Pharmacology and Therapeutics in 1929, and in 1935 honorary member of the British Pharmacological Society.

Regarding the personality of Straub, we have impressive portrayals by his pupils, e.g., by Gerhard Stroomann, later director of the Sanatorium Bühler Höhe, where Straub took a cure from former stroke-like disorders. All of them stated that Straub was a great teacher. His lectures were vivid and had been prepared with great care. He insisted that the experiments did function properly and were not cheated. He was convinced that personal witnessing of an experiment was of paramount importance for students, since he was an enthusiastic experimenter himself, a great photographer, and a good amateur painter. For many of his listeners, Straub’s lectures and demonstrations were often beautiful and unforgettable events (Stroomann 1960; Krayer and Reiter 1998).

His pupil, Hans Gremels who later obtained the chair in Marburg, characterized his teacher as a great cordial genius who provided scientific aid very gently. Thereby, he succeeded in producing satisfaction with his own work and the responsibility of his coworkers. He usually did not stimulate his assistants with a scientific theme but let them decide according to their own interests (Gremels 1947). Taken together, it is not surprising that the Institute of Pharmacology was named after Walther Straub at the initiative of Wolfgang Forth (see below).

Since 1904, Walther Straub was married to Dagny, née Lee from Norway; the couple had two children, Harald and Peter.

## August Wilhelm Forst

**Fig. 8:**
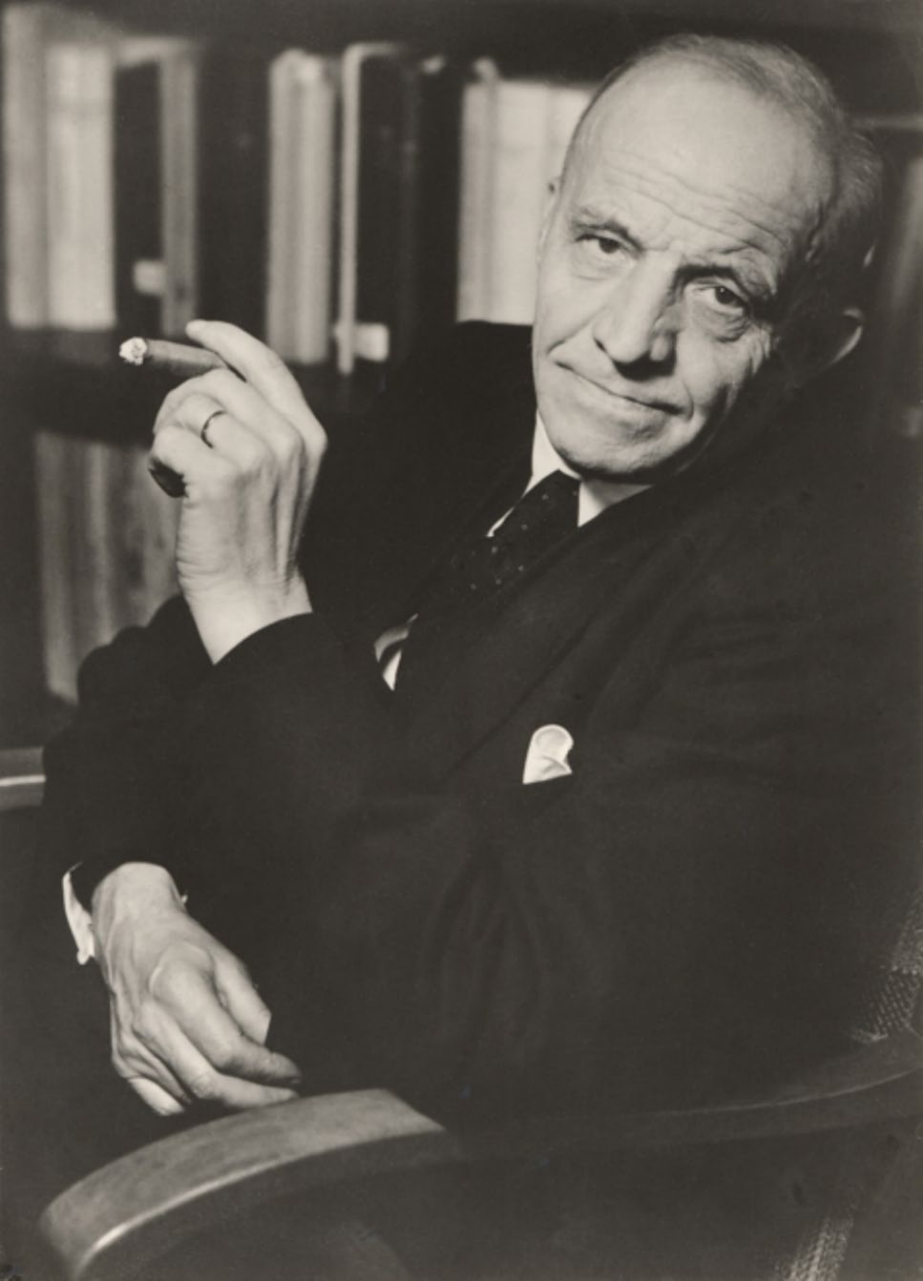
August Wilhelm Forst (about 1952) (Archive of Walther Straub Institute)

**Fig. 9:**
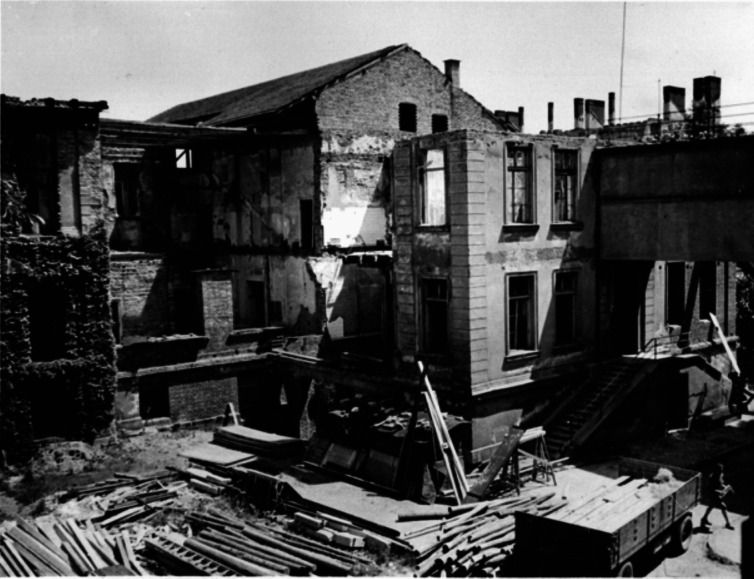
Ruins of the Institute of Pharmacology (May 1950) (Archive of Walther Straub Institute)

**Fig. 10:**
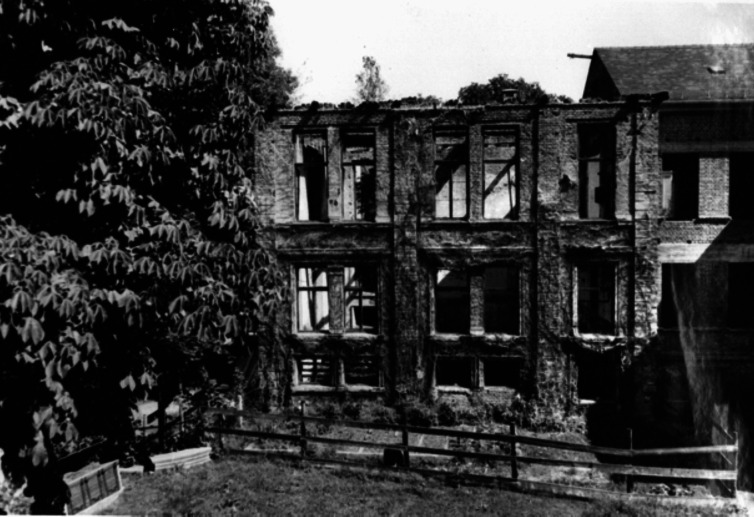
Former Lecture Hall that was torn down in 1954 (Archive of Walther Straub Institute)

After Walther Straub’s death, *Dr.med.* August Wilhelm Forst (Fig. 8) was commissioned to lead the Institute in the position of an Assistant, since the authorities refused to appoint him Professor of Pharmacology. Because of the Nuremberg Laws, he was forbidden to give lectures. Justice was done in 1946 when Forst was promoted to full professor and director of the Institute of Pharmacology in Munich. However, his reign was a heap of rubble rather that a building for science and teaching (Fig. 9) and it took more than a decade to provisionally repair parts of the building in Nussbaumstraße 26. Until 1959, the old part of the institute at Nussbaumstraße 28 could be partly used, but the lecture hall that remained for 10 years without a roof could not be saved and was torn down in 1954 (Fig. 10). Finally, the institute at Nussbaumstraße 26 was reconstructed, simple but appropriate to resume experimental work. The institute at Nussbaumstraße 28 was not renovated and was later substituted by a modern building (Forth and Klimmek 1994).

August Wilhelm Forst was born in Milano on June 10, 1890. He studied medicine from 1909 to 1914 in Heidelberg, Freiburg, and Munich and achieved the degree of *Dr.med.* in 1914 with a thesis on congenital varicose veins. During WW I, he served as a medical officer of the mountain regiment in Snowshoe Battalion and was awarded the Iron Cross 1st Class. Thereafter, he continued his studies in chemistry and was promoted to *Dr.phil*. under R. Willstätter in 1924 with a thesis on attempts to prepare beta oxindole propionic acid. Thereafter, he became an assistant of Walther Straub and qualified as a Lecturer in Pharmacology in 1928 with the thesis “On the detoxication of prussic acid” (Forst 1928). Herein, he showed that the toxic actions could be rapidly abolished by the intravenous injection of dioxyacetone followed by colloidal sulfur that was responsible for the biotransformation of cyanide to thiocyanate. Forst was engaged with the biological standardization of various drugs, including ergot alkaloids and opioids, and the active agents in the milk of *Lactuca virosa*, in the blossoms of *Arnica montana*, and in the hop cones of *Humulus lupulus*. To quantify sedative effects and spontaneous activity, Forst designed a suspended running plate (*Kippteller*) by which movements of small rodents could be registered on a kymograph (Forst 1938).

Besides, Forst wrote a comprehensive review on “Bismuth” (Forst 1935). Finally, he wrote a review on “Detoxication” (“*Entgiftung*”), a German analogue to R.T. William’s famous “Detoxication Mechanisms.” Unfortunately, the printing plates fell victim to the destruction by air raids shortly before the end of WW II (Forth and Klimmek 1994). Forst, after having retired, decided to write a completely new version that was published in 1966 (Forst 1966).

Forst together with his secretary Gertrud Sachse saved most books from the library when the institute burned down, was very busy in the reconstruction of the destroyed building, and served as Dean of the Medical Faculty from 1946 to 1948 and as Senator of the Ludwig Maximilian University from 1946 to 1956. In 1951, he was elected as a member of the Bavarian Academy of Sciences. In 1964, he was awarded the honorary doctorate of the Veterinary Faculty of Munich. August Wilhelm Forst retired in 1961 and passed away in Munich on August 4, 1981. He was married since 1925 to Caroline, née Weidert. The couple had one child, *Dr.rer.nat*. Dieter Forst.

## Manfred Kiese

**Fig. 11:**
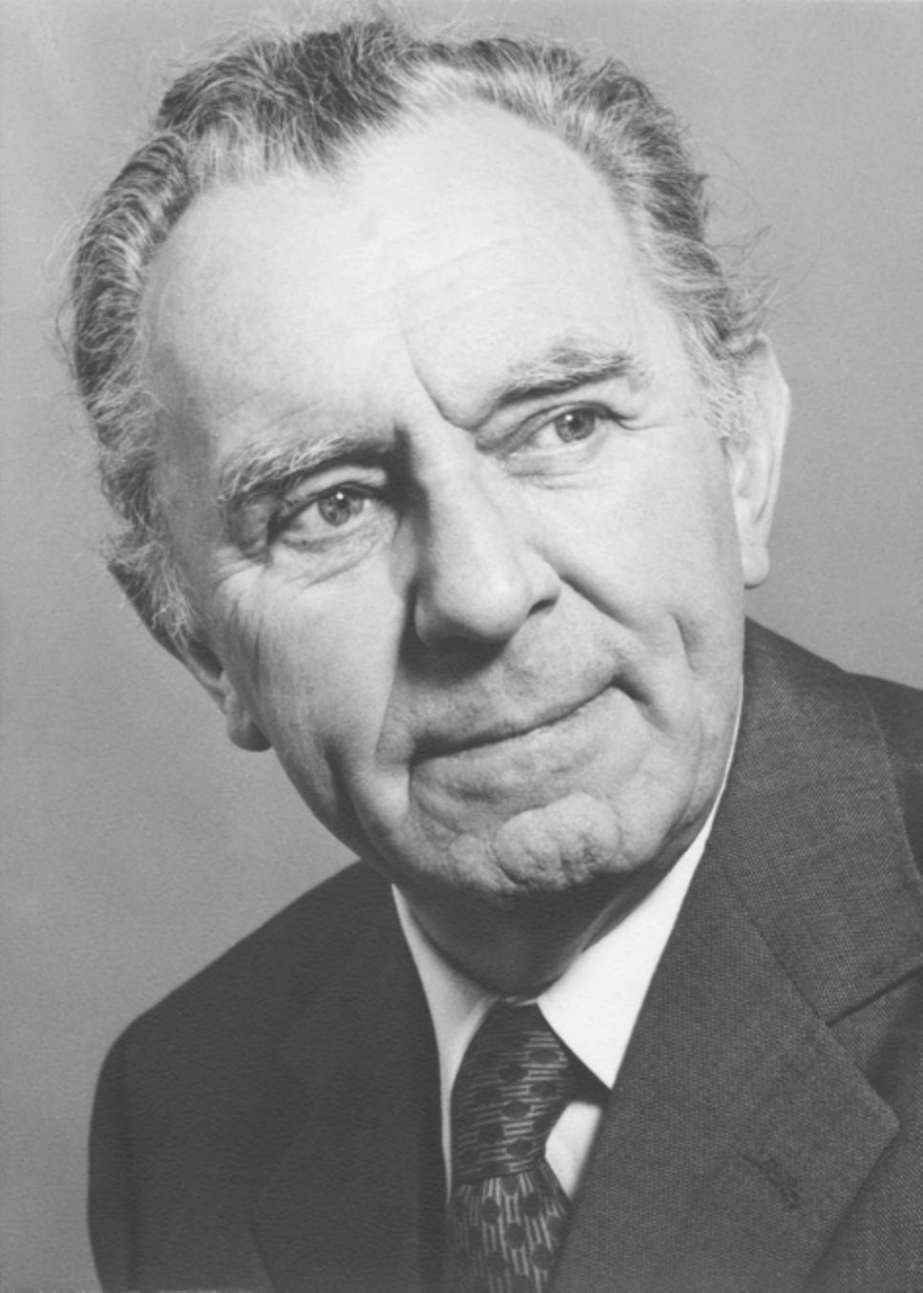
Manfred Kiese (about 1970) (Archive of Walther Straub Institute)

**Fig. 12:**
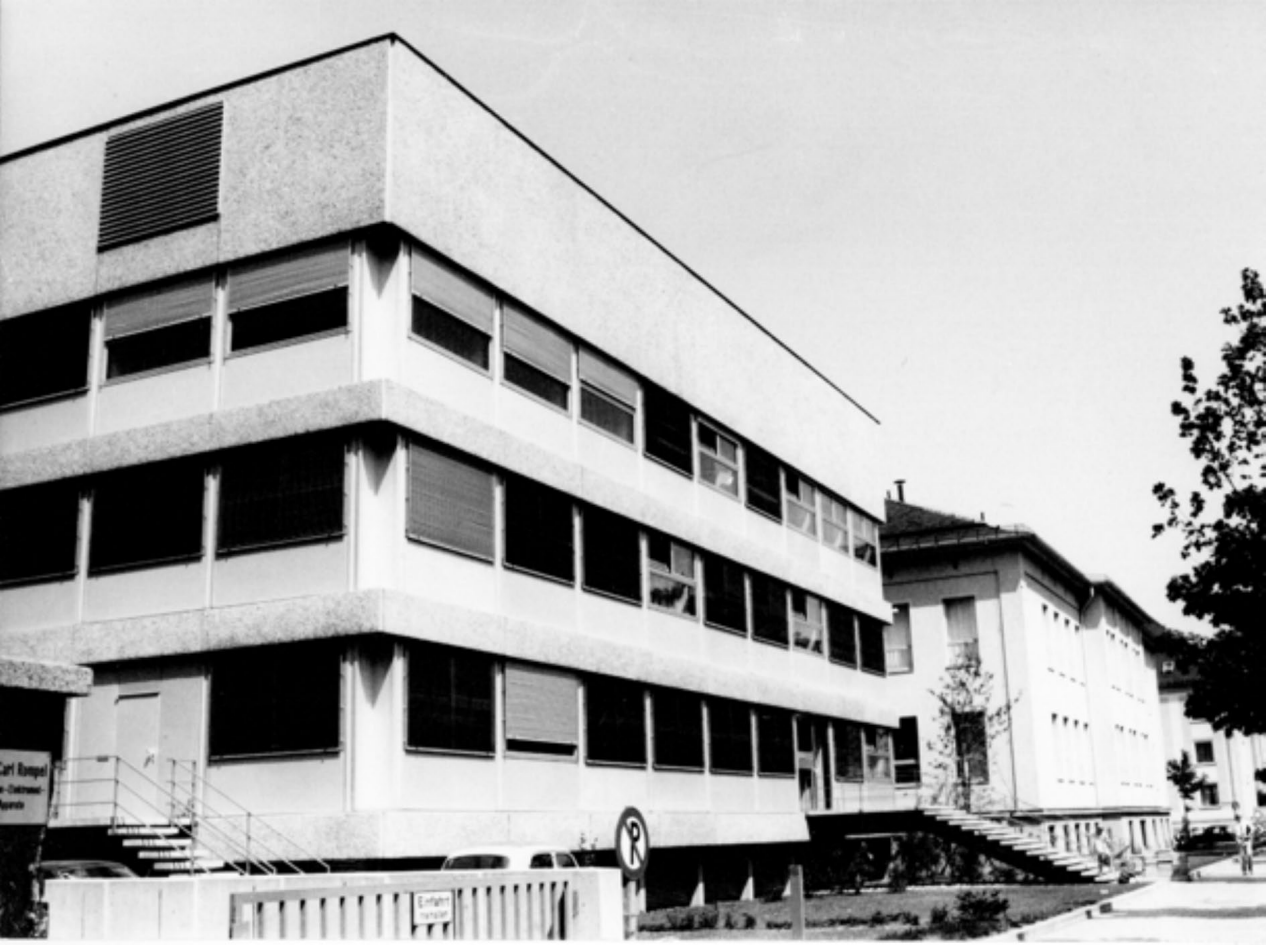
The newly constructed Institute of Pharmacology at Nussbaumstraße 28 (1969) (Archive of Walther Straub Institute)

Searching a suitable successor, the Medical Faculty favored a biochemically oriented pharmacologist and selected a pupil of Wolfgang Heubner, Manfred Kiese first choice who was heading the Institute of Pharmacology at Tübingen (Fig. 11). He obtained the call in 1960 that he accepted in 1961. He was accompanied with all but one assistant of Tübingen. Again, negotiations were extensive about a new building that should be erected on the place of the first institute at Nussbaumstraße 28. On the occasion of the topping-out ceremony, Kiese pointed out in his speech: “Pharmacology in Munich has suffered many decades from the first institute that had been erected between 1891 und 1893. When Walther Straub took the chair in 1923, he complained that the confusing construction plan was a source of constant worry and that the solidity of the building has prevented the justification of its pull down. Moreover, the spatial constriction by the later erected apartment houses does not allow the necessary enlargement of the complex” (Goerke 1972). Kiese continued: “In fact there had been serious considerations during my appointment to muddle around through the old building that had been ruined by the air raids.” Finally, in 1969, the new building could be occupied (Fig. 12) and the basement in Nussbaumstraße 26 was ready for remodeling. The main usable area of the new complex was around 4.500 m^2^ and the new lecture hall had a capacity of 420 seats. In addition, Kiese succeeded in his appointment negotiations to gather ample means for instrumental renewal and personnel increase. Hence, the Institute of Pharmacology in Munich graduated to the top ones in Germany.

Manfred Kiese was born on June 28, 1910, in Stettin, now Poland, and studied Medicine at the Universities of Hamburg, Frankfurt, Munich, and Berlin where he joined the Institute of Pharmacology (Wolfgang Heubner). In 1935, he was awarded the MD for “Pharmacological investigation of the smooth muscle of the lung, especially with some ephedrine-like substances.” Between 1935 and 1937, he was an assistant at the Institute of Pharmacology and studied the oxygen consumption of the dog’s heart in relation to work and the influence of drugs. In 1937, he became a Rockefeller Research Fellow at the Department of Biochemical Chemistry, Harvard University (Albert Baird Hastings) studying properties of the carbonic anhydrase. After his return to Heubner’s Institute, he continued the former studies and presented his Habilitation thesis on “The effects of carbon dioxide on the isolated guinea-pig ileum, and its sensitivity to drug actions and on isolated enzymes.” Probably owing to the beginning WW II, he turned his attention to the acute and chronic toxicity of explosives, in particular their effects on blood, which led him to study the mechanisms of ferrihemoglobin formation and its enzymic reduction that was augmented by several dyes. In addition, he was interested in the formation and properties of green hemoglobin derivatives (verdoglobins). These subjects found probably support due to war, since there were a lot of victims upon poisoning from explosives. Besides, Kiese collected the pertinent mostly Anglo-Saxon literature dealing with penicillin, a field he was not engaged experimentally. Most probably, the military authorities insisted in this topic to promote a German penicillin production because of the increasing wound infections that were no longer responsive to the formerly useful sulfonamides. Allegedly, this review (Kiese 1943) has been transmitted in 1943 to the allied Japan via a submarine by a coworker of the Japanese Embassy (Wolf 1999).

In 1945, the Berlin Institute was evacuated to the Agricultural School at Kappeln, north of Kiel, where Kiese found shelter with other colleagues from destroyed institutes of the University of Kiel. He continued his studies and published a series of 10 papers showing how phenylhydroxylamine was able to produce many equivalents of ferrihemoglobin in red cells, but not in solutions of hemoglobin. The involved enzymes, responsible for catalytic cycling, were analyzed what later became known as the “Kiese cycle.” Lectures for students were held on ships which were provided by the British occupation force, since the regular halls were destroyed.

In 1950, Kiese was appointed as full Professor of Pharmacology and the Head of the Institute at the University of Marburg, followed by his appointment at Tübingen in 1956. During this period, Kiese discovered first that aromatic amines were *N*-oxygenated in vivo by isolation of the corresponding nitroso compounds in the blood of dogs treated with arylamines. The formation of *N*-oxygenated aromatic amines was studied in isolated organs and microsomal fractions. When he became Director of the Institute of Pharmacology at the University of Munich in 1961, Kiese’s group extended studies on microsomal *N*- and *C*-oxidation of aromatic amines. Finally, he detected the unique property of 4-dimethylaminophenol to produce rapidly, but only a defined fraction of ferrihemoglobin in vitro and in vivo that made this hit-and-run drug suitable as an antidote in cyanide poisoning. Kiese is an author of 200 scientific papers, and his monograph “Methemoglobinemia, a Comprehensive Treatise” is the standard work on this topic comprising 2600 references (Kiese 1974). Recently, I had to be taught by the Editor that M. Kiese had published 47 original papers in this journal between 1947 and 1974 and was leading the count within this timeframe (Basol & Seifert 2024).

Manfred Kiese was a strict teacher for students and assistants, who relentlessly demanded conscientiousness, scientific honesty, and precise analytical thinking. I vividly remember his vehement combat of whitewashing when using the term “side effect” (“*Nebenwirkung*”) of a drug when an adverse effect was meant (“*unerwünschte Wirkung*”). Kiese impressed us with his extraordinarily broad scientific competence and experimental skills when he visited us on the bench almost daily and gave advice until his retirement in 1978.

On the contrary, Kiese refused working in committees, except for his short period as Dean of the Medical Faculty, and rarely visited events of the faculty or university. Besides being an enthusiastic scientist, Kiese fatherly lent his ear to the personal problems of his coworkers and gave wise advice.

Kiese retired in 1978 and died in 1983 in Munich. Since 1947, he was married to Edith, née Kunz who had passed 2 years before her husband. The couple had no own children. (For more details, the reader is referred to Eyer 2017b.)

## Wolfgang Forth

**Fig. 13:**
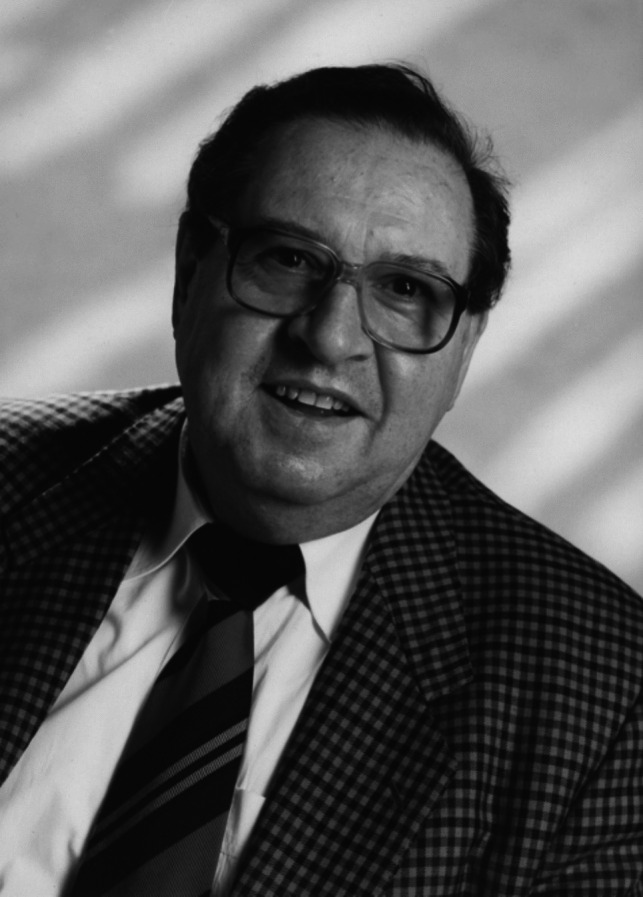
Wolfgang Forth (about 1992) (Photograph obtained from Forth’s family; Archive of Walther Straub Institute)

In 1980, Wolfgang Forth (Fig. 13) from the Ruhr-University in Bochum followed the call as director of the Pharmacological Institute of the Ludwig Maximilian University in Munich. In contrast to his predecessors, he had been saved from the experience of planning or restoring a new building but contended himself with planting magnolias in front of the Institute. Forth initiated the renaming of the Pharmacological Institute. On May 22, 1980, the Council of the Medical Faculty agreed that—in honor of Walther Straub—the institute was renamed into Walther Straub Institute of Pharmacology and Toxicology.

Wolfgang Forth was born in Mannheim on August 24, 1932, and studied medicine at the University of Heidelberg, where he was awarded the MD in 1958. The title of his thesis was: “A contribution to analysis of the centrally exciting actions of some sympathomimetic amines.” After his Medical Approbation in 1960, he entered the Institute of Pharmacology and Toxicology of the University of the Saarland in Homburg (W. Rummel). Here, his attention was turned to the movement of ions and water across the membranes of reticulocytes and intestinal mucosa cells in which he studied the effects of laxatives and cardiac glycosides. For these studies, he was awarded the Claude Bernard prize in 1966. In addition, his interests soon focused on the mechanisms of absorption of iron and chemically related metals in various segments of intestine, a scientific field for which he became best known. These studies led to his Habilitation thesis in 1967: “Studies on the intestinal absorption of iron and chemically related heavy metals in normal and anemic rats in vivo and in vitro. A contribution to the specificity of the mucosal iron-binding system.” In 1974, Forth was appointed Professor and Head of the Institute of Pharmacology and Toxicology at the University of Bochum, and in 1980, he became Director of the Institute of Pharmacology and Toxicology at the Ludwig Maximilian University of Munich, where Forth extended his studies to various metal–metal interactions during transport in the intestinal tract. Forth was an author of numerous scientific papers dealing with a broad spectrum of pharmacological and toxicological subjects. In addition, Forth has addressed many therapeutic problems in essays of *Deutsches Ärzteblatt*, whereby he became known to a broad medicinal public.

Finally, Forth was co-editor of *Pharmakologie und Toxikologie*, a textbook renowned in German-speaking countries since 1975 that is meanwhile in its 13^th^ edition, although with a changed team of editors.

Forth was a member of many committees and contributed to many discussions in the Medical Faculty. He had particularly high historical interests and literary passions, resulting in separate contributions such as in an Addendum “Building and Men” (Forth and Klimmek 1994) or “Men and Fungi” (Forth et al. 1997). In fact, he has told us that he had flirted at a young age of becoming a journalist.

Wolfgang Forth retired in the year 2000 and passed away on April 12, 2009, in Munich. He was married to Dagmar, née von Blomberg since 1959. The couple has two sons and two daughters. (For more details, the reader is referred to Eyer 2017a.)

## Peter Eyer

Although the appointment committee advertised the professorship early in 1998, a vacancy ensued with the retirement of Wolfgang Forth, because the first appointment negotiations were unsuccessful. In the meantime, I had been entrusted with the management of the chair, which unexpectedly dragged on for several years and was stressful for all coworkers.

I had been the head of the Department of Biochemical Toxicology and at that time mainly engaged in improving the treatment of organophosphate poisoning. In trying so, we analyzed the kinetic data of the single reactions involved, which allowed in silico simulations, validation in experimental models, and finally testing of antidotal strategies in poisoned patients in several clinical studies.

**Fig. 14:**
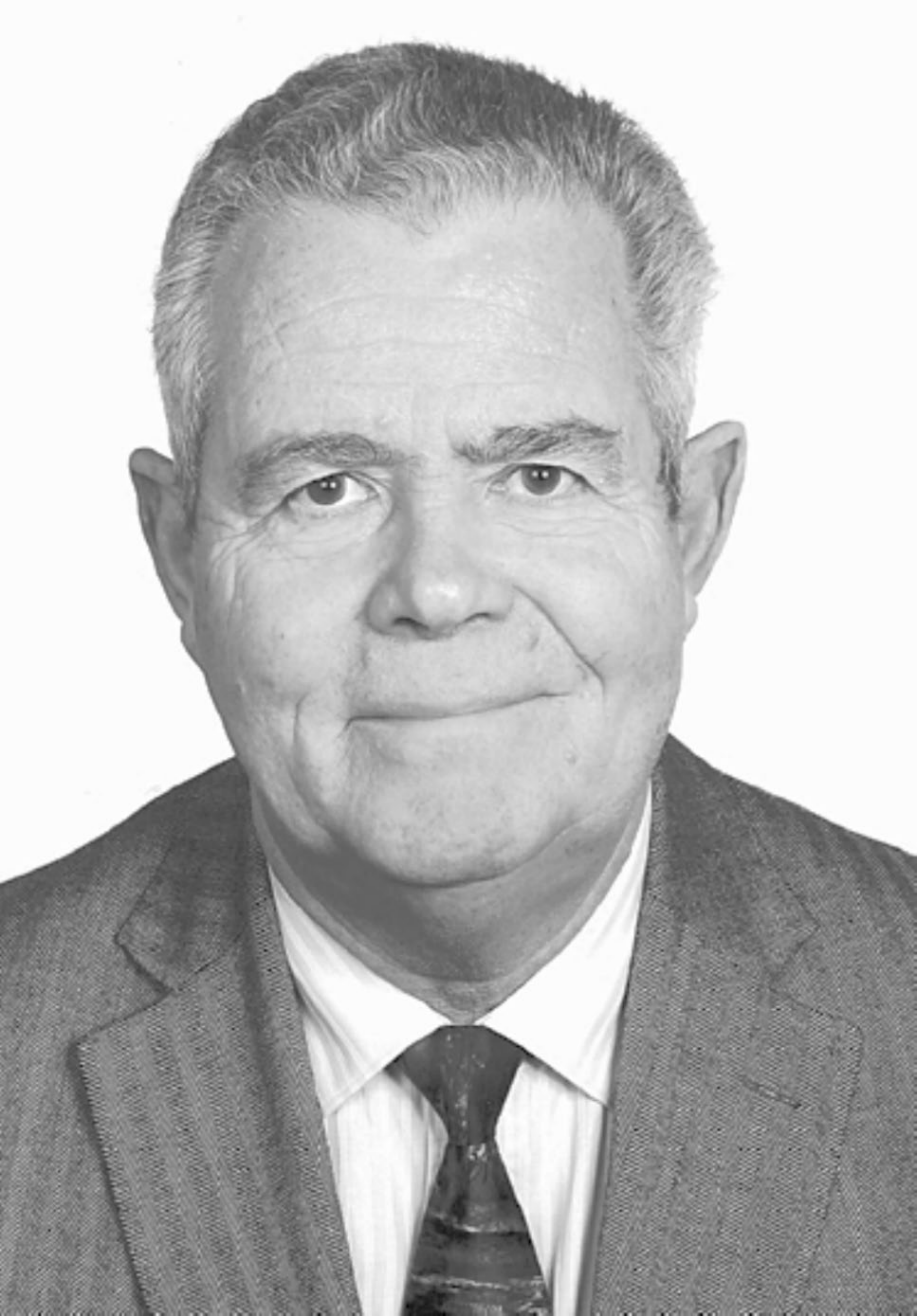
Peter Eyer (2015) (private)

Born on June 6, 1942, in Munich (Fig. 14), I studied medicine in Munich and Freiburg and was awarded the MD in 1968 in the group of Dirk Pette with an enzymological thesis: “Purification and properties of glucose-1-phosphate-kinase.” After my medical approbation in 1970, I was allowed to join Kiese’s group that was busy searching methemoglobin-forming aminophenols as an antidote against cyanide poisoning, of which 4-dimethylaminophenol was particularly useful in that it forms very rapidly methemoglobin to complex cyanide. The particular advantage of this antidote was caused by its hit-and-run kinetics, i.e., methemoglobin formation was complete within a few minutes and stopped rapidly at a given level. My task was to unravel the underlying mechanisms. It turned out that 4-dimethylaminophenol is co-oxidized by oxyhemoglobin to yield a phenoxyl radical that in turn oxidizes ferrohemoglobin to methemoglobin and is reduced back to the parent aminophenol. The catalytic cycle is terminated by disproportionation of the radical giving rise to the quinonimine that rapidly reacts with sulfhydryl groups of hemoglobin and glutathione within red cells. With part of these studies, I got my Habilitation in 1976 on “Biotransformation and actions of 4-dimethylaminophenol.”

Studies of various quinonimines and differently substituted nitrosoarenes with sulfhydryls revealed a variety of reaction pathways hitherto unknown in chemical literature but of major toxicological importance. I got the tenure professorship (C-2) in 1980 and (C-3) in 1998 at the same institute and headed the Walther Straub Institute temporarily from the year 2000 until my retirement in 2007. (For more details, the reader is referred to Eyer 2014.)

During this interregnum, several structural problems arose: Early in the 1990s, it was stated that the Walther Straub Institute did not meet the fire protection requirements, particularly of the new building that had been erected only some 25 years before. Moreover, the retirement of Forth gave the opportunity to reconsider the future focus of the chair. The majority of the Medical Faculty felt it appropriate that the chair was more intensely aimed at pharmacological issues and less in toxicological ones as in the last decades. In addition, the chair should be reduced in staff and consequently space. To this end, the chair was considered to move to a floor in the newly built Department of Chemistry and Pharmacy in Munich-Großhadern. This premature plan was impracticable, because several facilities were lacking, e.g., housings for experimental animals, controlled area for working with isotopes, and isotope storage facilities. In addition, the main usable area was less than 1/5 of the former. This “offer” deterred the first candidate and further negotiations were stopped. Next, there were plans to install a toxicological dependency at the old place in the heart of the city. Such a gap of 10 km would be impracticable to maintain a vivid corporate identity and was inacceptable for a presumptive successor.

**Fig. 15:**
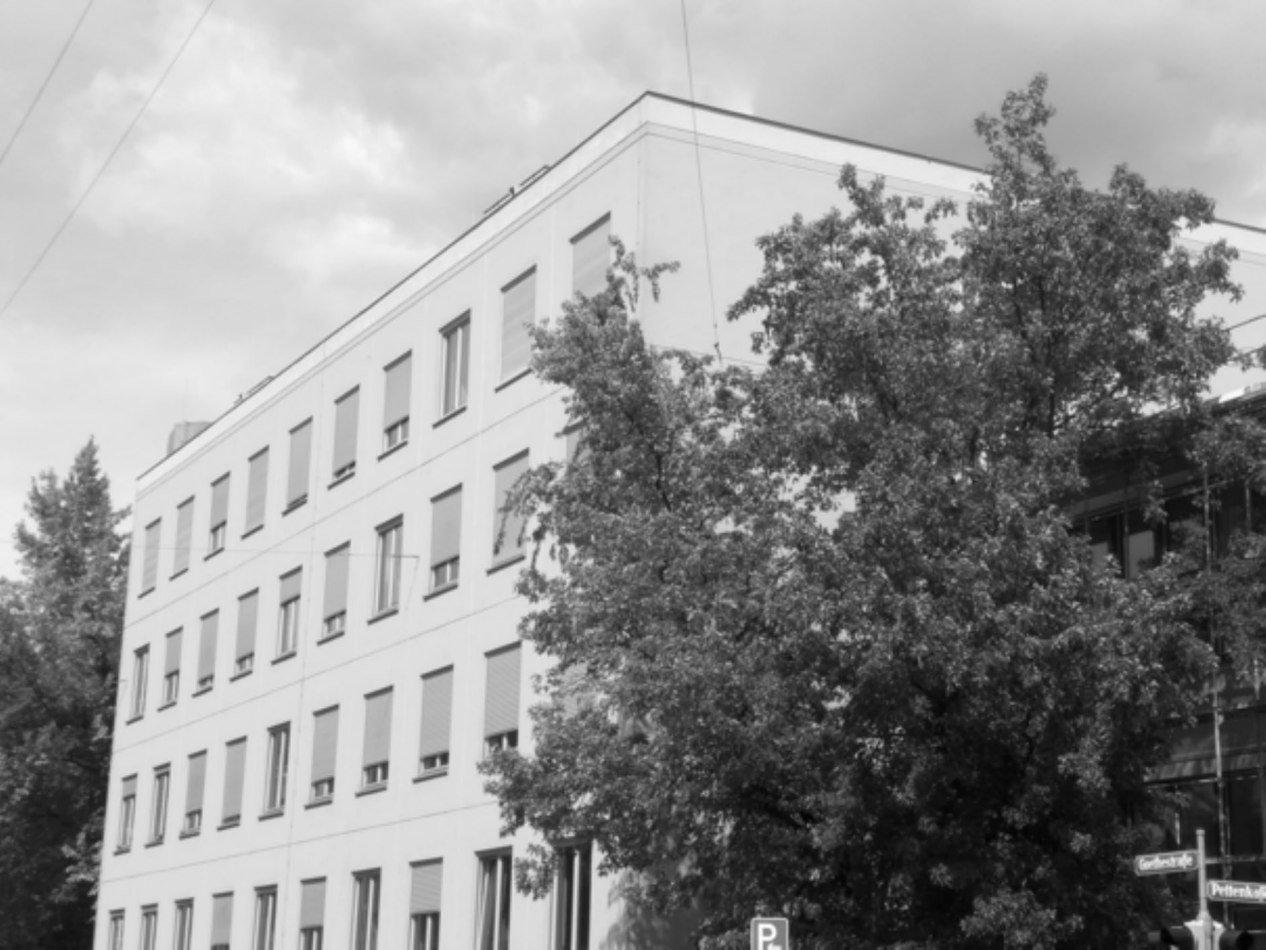
Walther Straub Institute at Goethestraße 33 (at present) (Archive of Walther Straub Institute)

Finally, the faculty agreed to establish the Walther Straub Institute in the completely renewed building of the former Institute of Physiological Chemistry in Goethestraße 33 that had moved to the campus of the Biomedical Center in Martinsried/Munich. In turn, the Institute of Legal Medicine should be situated in the renewed building at Nussbaumstraße, where a toxicological department of the Walter Straub Institute was hosted. The necessary renovation work was possible, and the staff of the Walther Straub Institute moved into the building at Goethestraße 33 in 2004 (Fig. 15).

New negotiations with an additional candidate dragged on because drastic austerity programs of the Bavarian government led to personnel cuts. Of course, a vacant chair is particularly helpless and the target of many desires. Reduced staff at high teaching commitments made the chair increasingly less attractive. Finally, the management of Ludwig Maximilian University and the active support by the Medical Dean, Dietrich Reinhard, succeeded in a third round of appointment negotiations and could convince Thomas Gudermann from Philipps University Marburg to accept. On May 1^st^, 2008, Prof. *Dr.med*. Thomas Gudermann took over the chair (For details, see Eyer & Gudermann 2011.)

## Thomas Gudermann

**Fig. 16:**
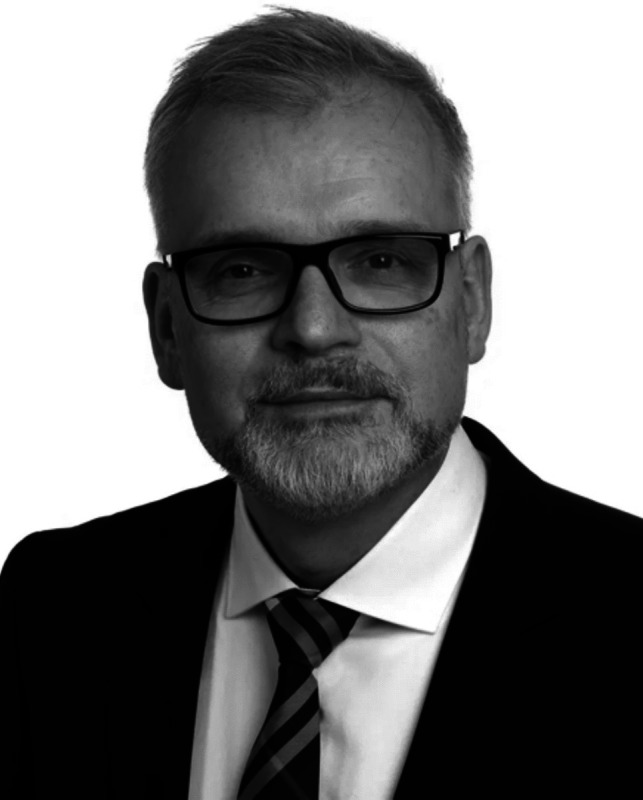
Thomas Gudermann, present Dean of Medical Faculty of Ludwig Maximilian University, Munich (LMU Klinikum 2021)

Born on December 7, 1960, in Lippstadt/Westfalen, Gudermann (Fig. 16) studied medicine in Münster and Ber Sheba, Israel, and was awarded the MD in 1989 with an experimental endocrinological work under Eberhard Nieschlag in Münster. After his Habilitation in 1998 in the Institute of Pharmacology at the FU in Berlin (Günter Schulz) on signal transduction of glycoprotein hormone receptors, he accepted the appointment of a full professorship at the Pharmacological Institute of the Philipps University of Marburg. After having obtained the chair in Munich, Gudermann started the conceptual, instrumental, and personnel reorganization of the institute, which comprises 17 working groups led by 7 professors and 3 lecturers.

In the last two decades, Gudermann’s research has focused on the role of transient receptor potential channels (TRP) in health and disease. Of the many TRPs, melastatin-like transient receptor potential channels (TRPM) are multifunctional cation channels that function as crucial cellular sensors involved in many physiological processes, including mineral homeostasis, blood pressure, cardiac rhythm, and immunity, as well as photoreception, taste reception, and thermoreception. Until now, eight family members have been defined (TRPM1-TRPM8). Of these, TRPM6 and TPRM7 are expressed in the kidney that control cellular and body homeostasis of the divalent cations, Zn^2+^, Mg^2+^, and Ca^2+^. The present intention of Gudermann’s group aims at elucidating the role of these TRPM channels in kidney pathology (Chubanov et al. 2024). Earlier, Gudermann’s group has shown that the intestinal TRPM7 is crucial for the homeostasis of the divalent cations in cultured intestinal cells (Mittermeier et al. 2019). From 2014 until now, Gudermann has been a spokesperson of the Transregional Collaborative Research Center TRR 152, “TRiPs to Homeostasis – Maintenance of Body Homeostasis by Transient Receptor Potential Channel Modules,” DFG.

Since 2021, Thomas Gudermann is Dean of the Faculty of Medicine, Ludwig Maximilian University Munich, and since 2024 Designated Chairman of the Executive Board, M1-Munich Medicine Alliance, which will pool the strengths of the member institutions of Munich as a center of medical excellence.

## Data Availability

No datasets were generated or analysed during the current study.
